# Tissue-specific metabolomic profiling after cardiopulmonary bypass in fetal sheep

**DOI:** 10.3389/fcvm.2022.1009165

**Published:** 2022-12-12

**Authors:** Wentao Wu, Yun Teng, Miao Tian, Bingxin Huang, Yuhang Deng, Huili Li, Haiyun Yuan, Jimei Chen, Xiaohong Li, Chengbin Zhou

**Affiliations:** ^1^Department of Cardiovascular Surgery, Guangdong Provincial Cardiovascular Institute, Guangdong Provincial People's Hospital, Guangdong Academy of Medical Sciences, Guangzhou, China; ^2^Guangdong Provincial Key Laboratory of South China Structural Heart Disease, Guangdong Provincial Cardiovascular Institute, Guangdong Provincial People's Hospital, Guangdong Academy of Medical Sciences, Guangzhou, China

**Keywords:** cardiopulmonary bypass, fetal sheep, metabolomics, tissue-specific, cardiac insufficiency

## Abstract

**Objective:**

Fetal cardiopulmonary bypass (CPB) is essential to fetal heart surgery, while its development is limited by vital organ dysfunction after CPB. Studying organ metabolism may help to solve this problem. The objective of this study was to describe the tissue-specific metabolic fingerprints of fetal sheep under CPB and to associate them with organ functions.

**Methods:**

Ten pregnant ewes at 90–120 days of gestation were randomly divided into two groups. The bypass group underwent a 1-h fetal CPB, whereas the control group underwent only a fetal sternotomy. During bypass, echocardiography, blood gases, and blood biochemistry were measured. After bypass, lambs were sacrificed, and tissues of the heart, liver, brain, kidney, and placenta were harvested. The metabolites extracted from these tissues were analyzed using non-targeted metabolomics based on liquid chromatography-mass spectrometry techniques.

**Results:**

All tissues except the placenta displayed significant metabolic changes, and the fetal heart displayed obvious functional changes. Fetal sheep that underwent CPB had common and tissue-specific metabolic signatures. These changes can be attributed to dysregulated lipid metabolism, altered amino acid metabolism, and the accumulation of plasticizer metabolism.

**Conclusion:**

Fetal CPB causes tissue-specific metabolic changes in fetal sheep. Studying these metabolic changes, especially cardiac metabolism, is of great significance for the study of fetal CPB.

## Introduction

Congenital heart disease has an incidence prevalence of about 1%, with complex congenital heart disease accounting for about 20% of cases (1). Some complex congenital heart diseases develop from relatively simple primary lesions. If surgical intervention can be performed in the fetal period, the transition to complication can be prevented, and the fetal heart can be allowed to develop again *in utero* (2). Fetal heart surgery requires safe and reliable cardiopulmonary bypass (CPB), and previous animal studies have confirmed the feasibility of fetal CPB (3–6).

However, vital organ dysfunction after fetal CPB, especially placental dysfunction (5) and cardiac insufficiency (3, 6), remains an Achilles' heel for experimental fetal cardiac surgery. The course of postnatal CPB is often accompanied by complex metabolic changes due to ischemia-reperfusion injury and systemic inflammatory response (7). The metabolic effects of fetal CPB may be more complex than those of postnatal CPB because the fetus itself is in a relatively hypoxic intrauterine environment. Therefore, it is urgent to describe the metabolic changes induced by fetal CPB, which may help to reveal the mechanisms of post-bypass organ dysfunction from a metabolic perspective.

With the rapid development of omics technology, metabolomics has been used to study CPB-related ischemia-reperfusion injury (8), acute kidney injury (9), and cerebral ischemic protection (10). This study established a fetal sheep model of CPB and used untargeted metabolomics to follow metabolic changes in the fetal heart, liver, brain, kidney, and placenta after bypass. We aimed to elucidate the tissue-specific metabolic changes and overall metabolic signature of fetal CPB and explore the relationship between organ function and metabolism.

## Materials and methods

### Animals

A total of 10 pregnant small-tail Han sheep at 90–120 days of gestation were randomly divided into a control group (*n* = 5) and a bypass group (*n* = 5). The bypass group received sternotomy and fetal CPB for 1 h, whereas the control group received only sternotomy. All animals received humane care in compliance with the Guide for the Care and Use of Laboratory Animals, recommended by the US National Institutes of Health. The experimental protocol was approved by the Ethics Review Committee for Animal Experimentation of Guangdong Provincial People's Hospital.

### Surgical procedures

Surgical preparation was performed as previously described (3). Fetal cannulation was performed using a 12 Fr straight-tip venous cannula (Medtronic) in the right atrial appendage, and a 6 Fr straight-tip arterial cannula (Medtronic) in the main pulmonary artery. Arterial and venous cannulas were connected to a standardized set of extracorporeal tubing for infants (Medos). A non-pulsatile centrifugal pump (Revolution 5, Sorin) was used as the power source, and a membrane oxygenator (HIITE 800LT, Medos) and the placenta were both used for oxygenation. Air was used as the source of gas for the oxygenator to simulate the physiological hypoxic state of fetal sheep. 150–200 mL of adult sheep blood containing 10 mg (1,250 IU) heparin was used to prime the pump circuit. The volume is dynamically adjusted to maintain the flow above 150–200 ml/kg/min during CPB.

### Echocardiography assessment and biochemical analysis of blood samples

Fetal sheep underwent CPB for 60 min. Echocardiography was monitored at three time points: T0, before CPB; T1, 30 min after starting CPB; T2, immediately after weaning off CPB. Several echocardiographic parameters were collected, including left ventricle Tei index (Tei-LV), right ventricle Tei index (Tei-RV), umbilical artery pulsatility index (UA-PI), and umbilical artery resistance index (UA-RI). The Tei index was calculated according to previous methods (33), using the formula (A-B)/B, where A was the interval between cessation and onset of mitral inflow (tricuspid inflow) and B was LV or RV ejection time. Fetal blood samples from the axillary artery and vein were also collected at T0, T1, and T2 for blood gas analysis and biochemical tests.

### Tissue collection

After bypass, the fetuses were euthanized. Fetal weight was measured, and the heart, liver, brain, kidney, and placenta were harvested. The harvested tissues of both groups were immediately flash-frozen in liquid nitrogen and stored at −80°C until metabolite extraction. The maternal sheep were resuscitated and returned to the farm.

### Metabolite extraction and ultrahigh performance liquid chromatography-MS/MS analysis

Tissue (100 mg) was ground separately in liquid nitrogen, and the homogenate was resuspended by vortexing with pre-cooled 80% methanol and 0.1% formic acid. Samples were incubated on ice for 5 min and then centrifuged at 15,000 g for 20 min at 4°C. A portion of the supernatant was diluted with liquid chromatography-mass spectrometry (LC-MS) grade water to a final concentration containing 53% methanol. The sample was then transferred to a new Eppendorf tube and centrifuged at 15,000 g, 4°C for 20 min. Finally, the supernatant was injected into a UHPLC-MS/MS system for analysis (11).

Quality control (QC) samples were prepared by mixing equal volumes of all 10 samples. Throughout the analysis, 3 QC samples were injected for every 10 samples to provide a set of data from which the stability and reproducibility of the method could be assessed.

UHPLC-MS/MS analysis was performed using a Vanquish UHPLC system and an Orbitrap Q ExactiveTM HF mass spectrometer. Samples were injected onto a Hypesil Gold column (100 × 2.1 mm, 1.9 μm) using a linear gradient of 17 min at a flow rate of 0.2 mL/min. The eluents for the positive polarity mode were eluent A (0.1% FA in water) and eluent B (methanol). The eluent for the negative polarity mode was eluent A (5 mM ammonium acetate, pH 9.0) and eluent B (methanol). The solvent gradients were set as follows. 2% B, 1.5 min; 2–100% B, 12.0 min; 100% B, 14.0 min; 100–2% B, 14.1 min; 2% B, 17 min. The Q ExactiveTM HF mass spectrometer was set to positive/negative polarity mode with a spray voltage of 3.2 kV, capillary temperature of 320°C, sheath gas flow rate of 40 arb, and aux gas flow rate of 10 arb.

### Data processing and analysis of LC-MS

The raw data files generated by UHPLC-MS/MS were processed using Compound Discoverer 3.1 for peak alignment, peak extraction, and quantification. The main parameters were set as follows: retention time tolerance of 0.2 min; actual mass tolerance of 5 ppm; signal intensity tolerance of 30%; signal/noise ratio of 3; and minimum signal intensity of 100,000, after which the peak intensities were normalized to the total spectral intensity. The normalized data was used to predict molecular formulas based on additive ions, molecular ion peaks, and fragment ions. The peaks were then matched against the mzCloud, mzVault, and MassList databases to obtain accurate qualitative and relative quantitative results. These metabolites were then annotated using the KEGG, HMDB, and Lipidmaps databases.

Metabolomic statistical analysis was performed using MetaboAnalyst 5.0, a comprehensive web-based metabolomics analysis tool (12). Relative peak intensities were initially log-transformed and then auto-scaled. Statistical significance (*P*-value) was calculated by *t*-test. Multivariate principal component analysis (PCA) and orthogonal partial least squares discriminant analysis (OPLS-DA) were used to observe data variance, and the value of variable importance in projection (VIP) for each metabolite was derived from the OPLS-DA model. The metabolites with VIP > 1 and a *P* < 0.05, fold change (FC) ≥ 1.5 or FC ≤ 0.67 were considered as differential metabolites. Finally, enrichment analysis was performed to evaluate the impact of individual metabolite alteration on different metabolic pathways, and *P* < 0.05 was considered significant.

### Statistical analysis of animal data

Shapiro-Wilk test was used to check normal distribution. The normally distributed data is presented as mean ± standard deviation. The student's *t*-test was used to compare pre-bypass fetal data between the two groups, and repeated-measures ANOVA was used to compare the two groups at other time points. Paired *t*-tests were used for within-group comparisons. When data was not normally distributed, the nonparametric Kruskal-Wallis test was used. *P* < 0.05 indicated statistically significant differences. Statistical analysis was performed with IBM SPSS Statistics 26.

## Results

### Effects of fetal CPB on vital organ functions

Fetal sheep CPB models were successfully established in all animals of the bypass group. There was no significant difference in fetal weight between the bypass and control groups (2.10 ± 0.59 kg vs. 1.49 ± 0.86 kg, *P* = 0.232). The echocardiographic data is shown in Table 1. The Tei indices of both ventricles in the bypass group increased during the bypass (T2 vs. T0, *P* < 0.05) and were higher than in the control group (*P* < 0.05). Heart rate in the bypass group decreased during bypass (T1 vs. T0, *P* = 0.027) and decreased much more than in the control group (*P* = 0.005). Other parameters, including UA-PI, and UA-RI, did not differ significantly between the two groups.

**Table 1:** Changes of hemodynamic parameters in fetal sheep during CPB.

	**Control**			**Bypass**			***P*-value**
	**T0**	**T1**	**T2**	**T0**	**T1**	**T2**	
Tei-LV	0.37 ± 0.09	0.45 ± 0.19	0.52 ± 0.12	0.35 ± 0.06	-	1.68 ± 0.54^*^	0.032
Tei-RV	0.32 ± 0.02	0.39 ± 0.06	0.43 ± 0.07	0.56 ± 0.13	-	1.41 ± 0.17^*^	0.002
UA-PI	1.35 ± 0.46	1.32 ± 0.38	1.36 ± 0.43	1.07 ± 0.39	1.23 ± 0.22	1.25 ± 0.31	0.598
UA-RI	0.77 ± 0.18	0.75 ± 0.24	0.79 ± 0.23	0.65 ± 0.14	0.70 ± 0.11	0.72 ± 0.11	0.575
HR (bpm)	148.67 ± 15.28	133.67 ± 14.84	134.67 ± 10.02	138.50 ± 21.33	88.00 ± 11.17^*^	74.25 ± 30.50	0.005

^*^P < 0.05 compared to T0.

T0, before CPB; T1, 30 min after starting CPB; T2, immediately after weaning off CPB.

LV, left ventricle; RV, right ventricle; UA-PI, umbilical artery pulsation index; UA-RI, umbilical artery resistance index; HR, heart rate.

As shown in Table 2, the PH values in the bypass group increased during bypass (*P* = 0.021 for T1 compared to T0, *P* = 0.031 for T2 compared to T0), but there was no significant difference in PH values between the two groups. Carbon dioxide (pCO2) levels in the bypass group showed a decreasing trend, significantly different from the increasing trend in the control group (*P* = 0.0004). Lactate values in the bypass group increased during bypass (T1 vs. T0, *P* = 0.008; and T2 vs. T0, *P* = 0.018) and were significantly different from those in the control group (*P* = 0.004). Changes in liver and kidney function are also shown in Table 2, with no significant differences between the two groups.

**Table 2:** Changes in blood gases and blood biochemical parameters of fetal sheep during CPB.

	**Control**			**Bypass**			***P*-value**
	**T0**	**T1**	**T2**	**T0**	**T1**	**T2**	
PH	7.24 ± 0.06	7.32 ± 0.10	7.27 ± 0.09	7.20 ± 0.06	7.34 ± 0.07^*^	7.32 ± 0.06^*^	0.796
PO_2_ (mmHg)	23.00 ± 3.61	25.33 ± 1.15	20.33 ± 1.15	20.00 ± 2.55	24.60 ± 8.44	32.60 ± 16.16	0.576
PCO_2_ (mmHg)	67.43 ± 3.06	75.50 ± 15.00	86.73 ± 6.92^*^	75.38 ± 12.95	36.60 ± 10.74^*^	28.88 ± 10.05^*^	0.0004
Lac (mmol/L)	1.98 ± 0.61	1.56 ± 0.80	1.89 ± 0.59	2.19 ± 0.76	6.53 ± 1.72^*^	7.58 ± 3.10^*^	0.004
ALT (U/L)	17.33 ± 5.51	15.67 ± 5.69	12.00 ± 3.46	12.75 ± 3.86	17.50 ± 5.45	22.25 ± 4.86^*^	0.423
AST (U/L)	24.00 ± 6.25	47.00 ± 16.82	50.00 ± 14.00	23.00 ± 8.04	54.50 ± 30.84	48.00 ± 24.09	0.912
BUN (mmol/L)	9.83 ± 1.96	9.93 ± 1.33	10.90 ± 1.39	10.76 ± 2.33	10.55 ± 2.67	10.54 ± 2.62	0.807
Cr (μmol/L)	89.00 ± 45.31	93.33 ± 39.72	103.33 ± 47.52	98.50 ± 39.52	114.50 ± 67.22	107.00 ± 60.92	0.755

^*^P < 0.05 when compared to T0.

T0, before CPB; T1, 30 min after starting CPB; T2, immediately after weaning off CPB.

PO_2_, partial pressure of oxygen; PCO*2*, partial pressure of carbon dioxide; Lac, lactic acid; ALT, alanine aminotransferase; AST, aspartate aminotransferase; BUN, blood urea nitrogen; Cr, creatinine.

### Metabolic changes in the heart

**Figure 1:**
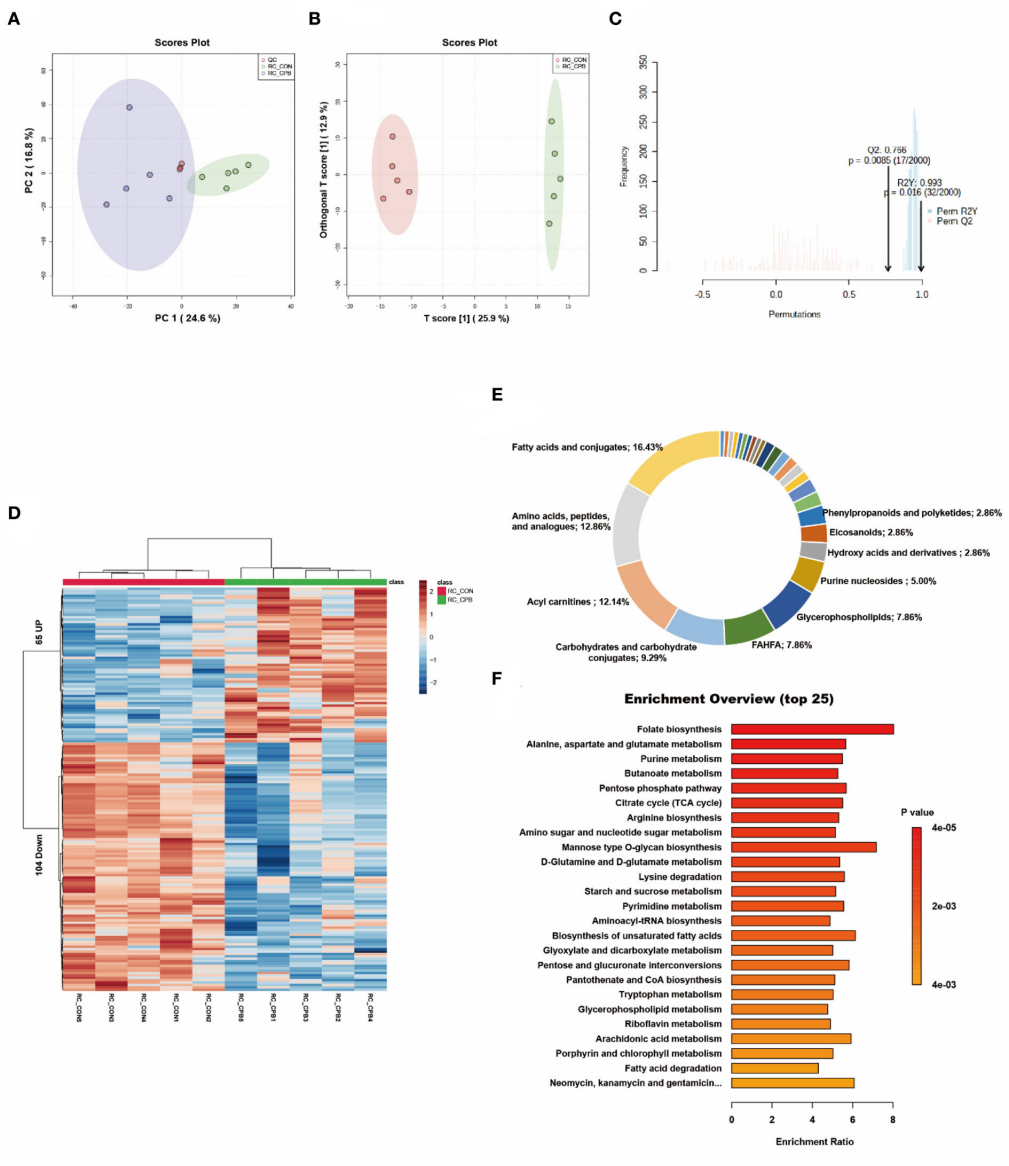
Metabolic fingerprints of the heart. **(A)** PCA score plots of bypass and control heart samples; **(B)** OPLS-DA score plots of bypass and control heart samples. **(C)** Permutation test of the OPLS-DA model; **(D)** Heat map of metabolite alterations. Red bands indicate upregulation of metabolite levels, while blue bands indicate downregulation of metabolite levels in the CPB group compared to the CON group; **(E)** Biochemical classification of altered metabolites; **(F)** Pathway enrichment analysis of altered metabolites.

PCA is an unsupervised data analysis method that generally captures the metabolic differences between different groups of samples and the degree of variation within each group. The PCA score plots demonstrated clear clustering between the two groups (Figure 1A). The QC samples were clustered in the middle area of the tested samples, indicating good reproducibility and stability of the metabolome workflow. A supervised OPLS-DA was performed to maximize the separation of the two groups. As shown in Figure 1B, the cardiometabolic fingerprints of these fetuses were separated. Also, the potential overfitting of the OPLS-DA model was assessed by two thousand permutation tests (Figure 1C), where R2Y = 0.993 (*P* = 0.016) and Q2 = 0.766 (*P* = 0.0085), indicating that the OPLS-DA model is highly stable and predictable. Using VIP > 1.0, FC > 1.5 or FC < 0.667, and *P* < 0.05 as thresholds, 169 differential metabolites were identified in the bypass group compared to the control group (Supplementary Table 1). The characteristics of these alterations were clustered, and the heatmap showed significant differential enrichment clusters between the two groups (Figure 1D). To better understand the molecular nature of these metabolites, we classified them according to their biochemical classes. The main categories identified were fatty acids and conjugates (16.43%), amino acids, peptides, and analogs (12.86%), and acylcarnitines (12.14%; Figure 1E). Long-chain polyunsaturated fatty acids (LCPUFAs), including docosahexaenoic acid (DHA) and docosapentaenoic acid (DPA), were reduced in the bypass group. Various acylcarnitines, usually used as fatty acid transporters, also showed significant changes. In comparison to the control group, glycogenic amino acids (methionine, glutamate, and threonine) were reduced, while intermediates of glucose metabolism (glucose 6-phosphate, fructose 6-phosphate) and the TCA cycle (succinic acid, ketoglutaric acid, cis-aconitic acid) were increased. Finally, we enriched the metabolic pathways of these altered metabolites. Glucose metabolism (TCA cycle, pentose phosphate pathway), amino acid metabolism (alanine/aspartate/glutamate metabolism, arginine biosynthesis), and lipid metabolism (butanoate metabolism, biosynthesis of unsaturated fatty acids) were the main affected pathways (Figure 1F).

### Metabolic changes in the liver

**Figure 2:**
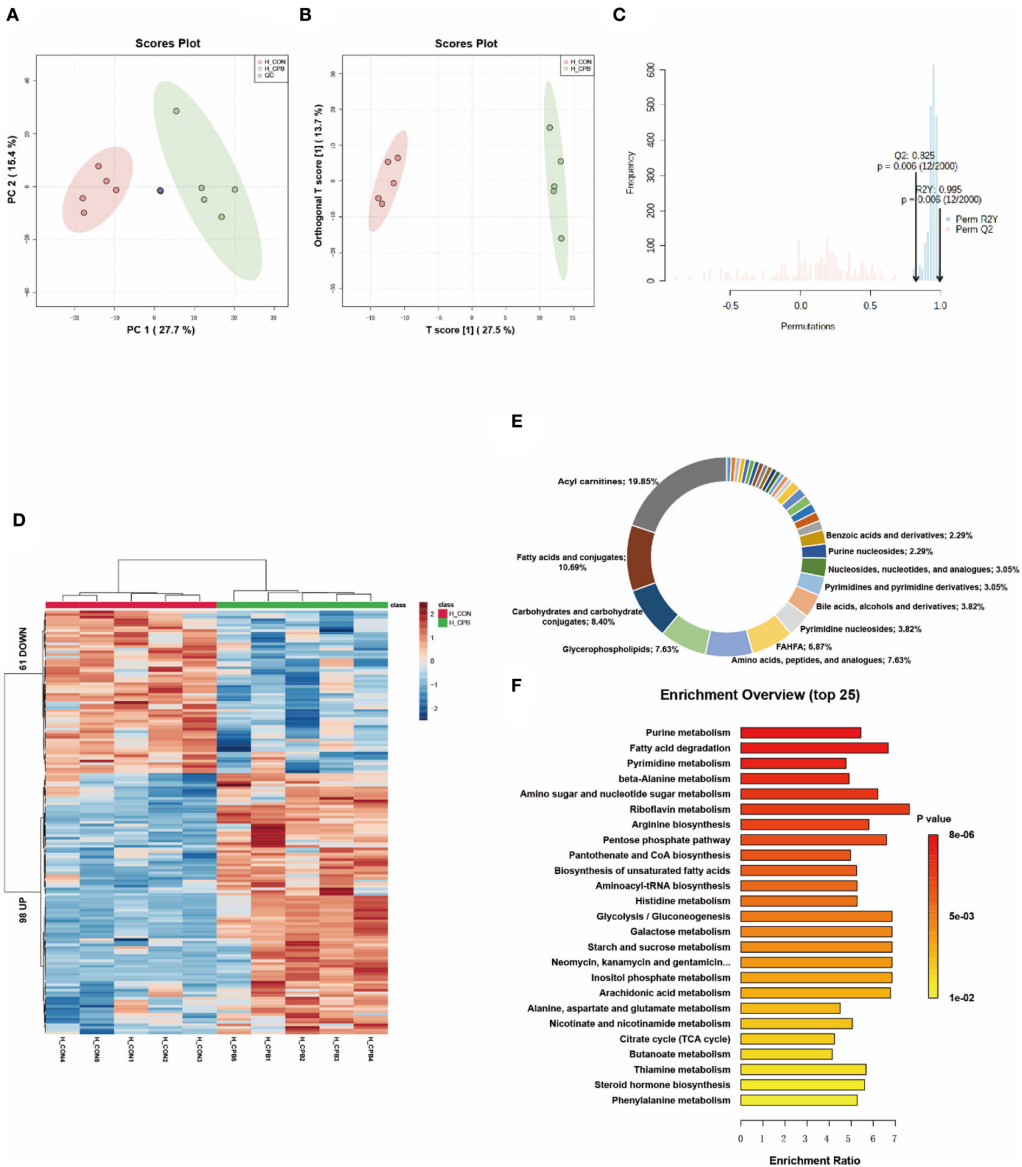
Metabolic fingerprints of the liver. **(A)** PCA score plots of bypass and control liver samples; **(B)** OPLS-DA score plots of bypass and control liver samples; **(C)** Permutation test of the OPLS-DA model; **(D)** Heat map of metabolite alterations. Red bands indicate upregulation of metabolite levels, while blue bands indicate downregulation of metabolite levels in the CPB group compared to the CON group; **(E)** Biochemical classification of altered metabolites; **(F)** Pathway enrichment analysis of altered metabolites.

Multivariate analysis revealed significant differences in liver metabolism between the bypass and control groups (Figures 2A–C). We identified 159 significantly altered metabolites, which could be well clustered (Figure 2D and Supplementary Table 2). Among these altered metabolites, the primary classifications included acylcarnitines (19.85%), fatty acids and conjugates (10.69%), and carbohydrates and carbohydrate conjugates (8.40%; Figure 2E). Enrichment analysis revealed a high enrichment of pathways of nucleotide metabolism (purine metabolism, pyrimidine metabolism) and lipid metabolism (fatty acid degradation, unsaturated fatty acid biosynthesis; Figure 2F). The NAD+, FAD, and NADH levels were elevated in the bypass group, indicating increased energy metabolism. Bile acids (glycocholic acid, taurochenodeoxycholic acid) were increased, whereas fatty acids were decreased, suggesting that bile acid metabolism and fatty acid metabolism were promoted during the bypass. In addition, mono-ethylhexyl phthalic acid (MEHP), an *in vivo* metabolite of a hepatotoxic plasticizer, was observed to accumulate in the bypass group (136.7-fold of the control group, *P* = 0.00003).

### Metabolic changes in the kidney

**Figure 3:**
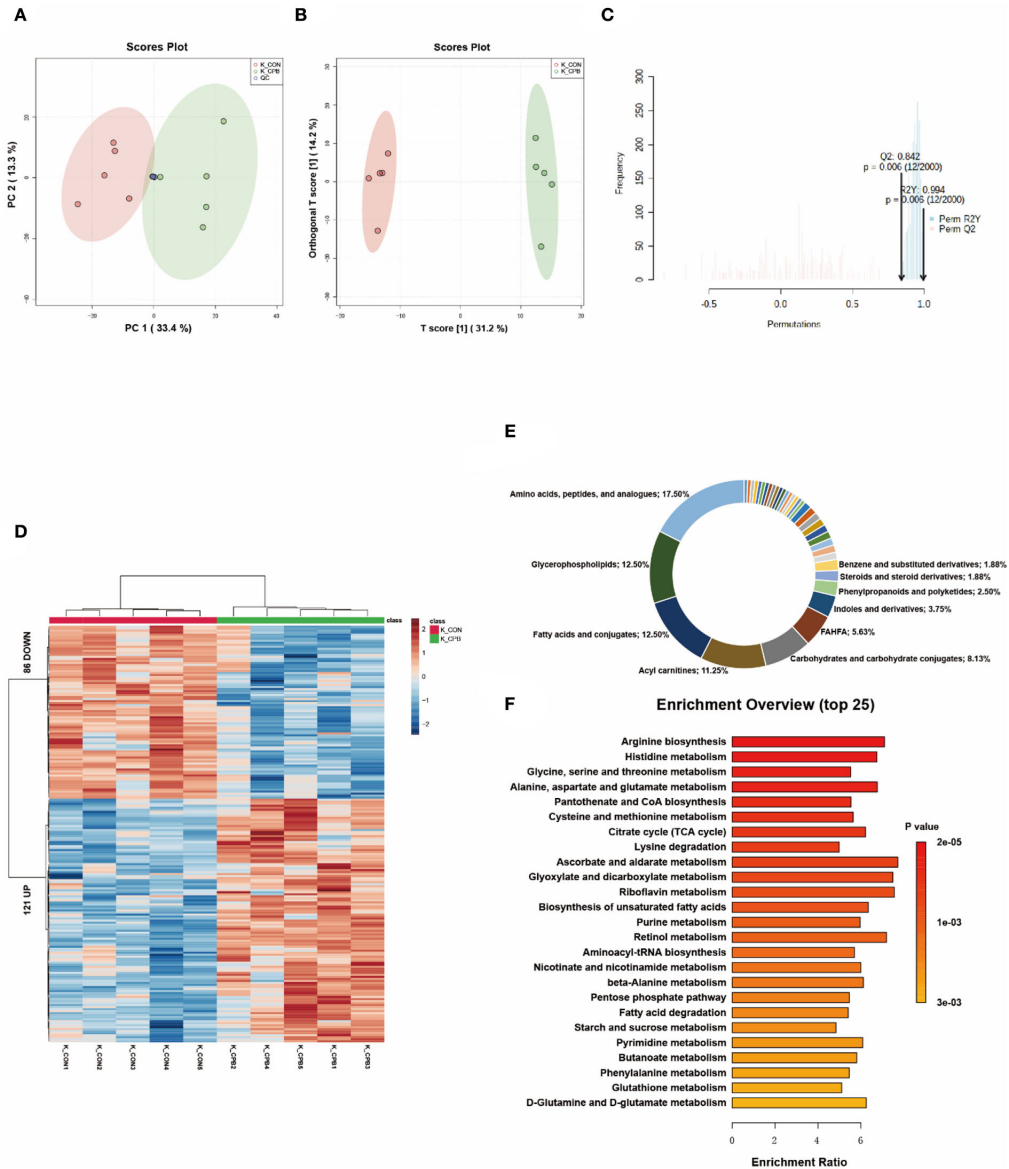
Metabolic fingerprints of the kidney. **(A)** PCA score plots of bypass and control kidney samples; **(B)** OPLS-DA score plots of bypass and control kidney samples; **(C)** Permutation test of the OPLS-DA model; **(D)** Heat map of metabolite alterations. Red bands indicate upregulation of metabolite levels, while blue bands indicate downregulation of metabolite levels in the CPB group compared to the CON group; **(E)** Biochemical classification of altered metabolites; **(F)** Pathway enrichment analysis of altered metabolites.

The metabolites of the kidney can be divided into two distinctive groups in the score plots (Figures 3A–C). The heatmap shows that the kidney displayed the greatest variation in metabolite abundance compared to other tissues (Figure 3D). A total of 207 differential metabolites were identified (Supplementary Table 3), with amino acids, peptides, and analogs (17.50%), glycerophospholipids (12.50%), fatty acids, and conjugates (12.50%) accounting for the major proportion (Figure 3E). Among the top 10 enriched pathways, 60% involved amino acid metabolism (Figure 3F), indicating the important role of amino acid metabolism in the bypass kidney. Changes in renal energy metabolism were characterized by increased mitochondrial metabolism (TCA cycle, oxidative phosphorylation) and fatty acid metabolism (biosynthesis of unsaturated fatty acids, degradation of fatty acids).

### Metabolic changes in the brain

**Figure 4:**
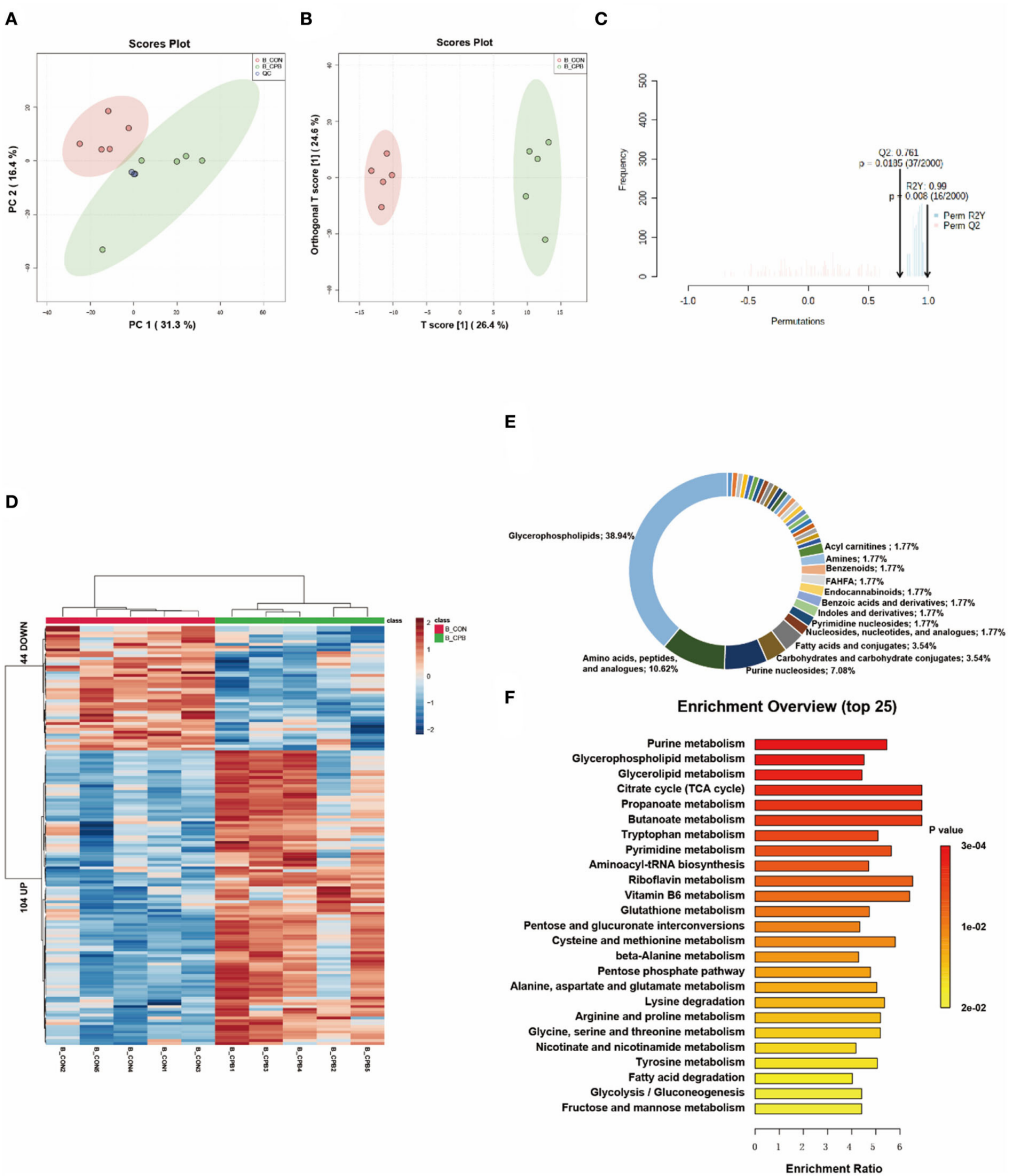
Metabolic fingerprints of the brain. **(A)** PCA score plots of brain samples from the bypass and control groups; **(B)** OPLS-DA score plots of brain samples from the bypass and control groups; **(C)** Permutation test of the OPLS-DA model; **(D)** Heat map of metabolite alterations. Red bands indicate upregulation of metabolite levels, while blue bands indicate downregulation of metabolite levels in the CPB group compared to the CON group; **(E)** Biochemical classification of altered metabolites; **(F)** Pathway enrichment analysis of altered metabolites.

Multivariate analysis revealed significant metabolic differences between the brains of the bypass and control groups (Figures 4A–C). One hundred and forty eight differential metabolites were identified (Figure 4D and Supplementary Table 4). Most of these metabolites could be classified as glycerophospholipids (38.94%), amino acids, peptides, and analogs (10.62%), and purine nucleosides (7.08%; Figure 4E). The pathways of glycerophospholipid metabolism, nucleotide metabolism, and amino acid metabolism were highly enriched (Figure 4F). In addition, metabolic changes associated with oxidative stress damage were also observed. Carnosine can scavenge reactive oxygen species (ROS) formed by lipid peroxidation (13), which is reduced in the bypassed brain (0.49-fold of the control group, *P* = 0.032). Palmitoylethanolamide (PEA), a lipid that accumulates during cellular stress (14), is increased in bypassed brains (2.30-fold of the control group, *P* = 0.014).

### Metabolic changes in the placenta

**Figure 5:**
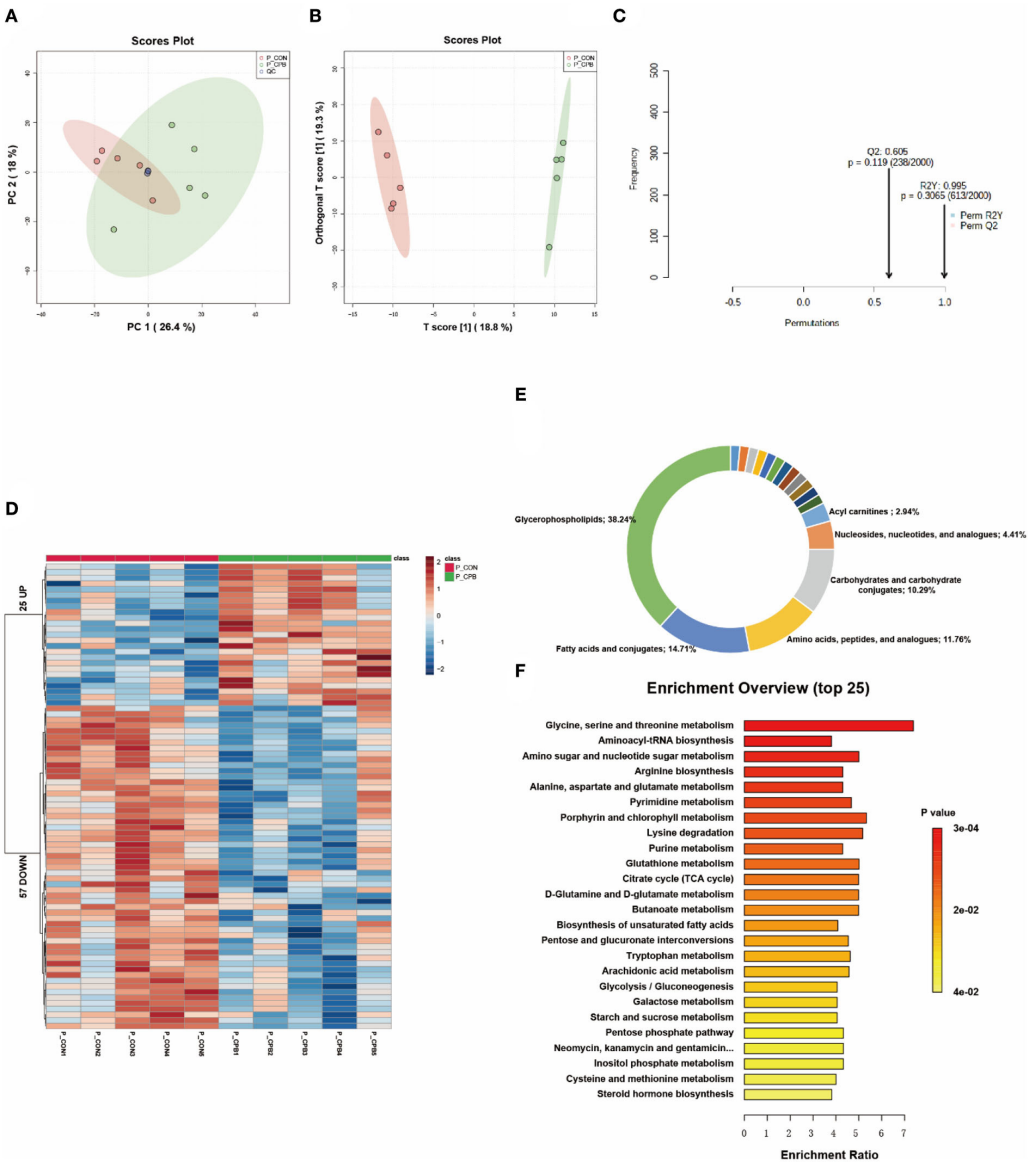
Metabolic fingerprints of the placenta. **(A)** PCA score plots of bypass and control placenta samples; **(B)** OPLS-DA score plots of bypass and control placenta samples; **(C)** Permutation test of the OPLS-DA model; **(D)** Heat map of metabolite alterations. Red bands indicate upregulation of metabolite levels, while blue bands indicate downregulation of metabolite levels in the CPB group compared to the CON group; **(E)** Biochemical classification of altered metabolites; **(F)** Pathway enrichment analysis of altered metabolites.

Placenta was the only tissue that could not be clearly distinguished by metabolomics. In the PCA score plot, the 95% confidence intervals of the two groups overlapped (Figure 5A). Although OPLS-DA could separate the two groups, the permutation test displayed overfitting of the OPLS-DA model (Figures 5B,C). Using FC > 1.5 or FC < 0.667 and *P* < 0.05 as thresholds, 82 metabolites were identified as differential (Figure 5D and Supplementary Table 5). The main categories of these metabolites included glycerophospholipids (38.24%), fatty acids and conjugates (14.71%), and amino acids, peptides, and analogs (11.76%; Figure 5E). The pathways of amino acid metabolism, nucleotide metabolism, and unsaturated fatty acid metabolism were highly enriched (Figure 5F). In terms of oxidative stress, betaine and ascorbate palmitate were found to increase in the bypass group. In addition, many phospholipids were significantly reduced in the bypass placenta.

## Discussion

Previous studies on fetal CPB have proved that it was technically feasible to place the fetal sheep on and off bypass (3–6). However, most lambs died in several hours after CPB because of placental dysfunction, characterized by elevated placental vascular resistance and deterioration of fetal gas exchange (5). Moreover, even though placental dysfunction was prevented, the fetuses still suffered from cardiac insufficiency after bypass (6). These studies clearly demonstrate that the major limitation to successful clinical fetal cardiac surgery would not be technical but associated with the complex pathophysiologic responses of the fetus to various interventions.

Metabolomics is a relatively young branch of “omics” science and has been applied to several studies on neonatal and infant cardiac surgery. Correia et al. demonstrated substantial shifts in the metabolic profile of infants after cardiac surgery, including changes in ketone bodies and lipid metabolism (34). Davidson et al. proved CPB could induce age-independent metabolic changes in neonates and infants, characterized by a progressive global deficiency in amino acid levels (35). However, no studies have been published on metabolic changes induced by fetal CPB.

This report described the impact of fetal CPB on organ metabolism and function. Significant metabolic changes occurred in almost all tissues (except the placenta), and the fetal heart displayed obvious functional changes. Enrichment analysis revealed tissue-specific metabolic fingerprints as well as common metabolic signatures among organs. Lipid metabolism and amino acid metabolism were the most affected pathways. In addition, accumulation of plasticizer metabolites was observed in several tissues.

### Cardiac metabolism and cardiac function

**Figure 6:**
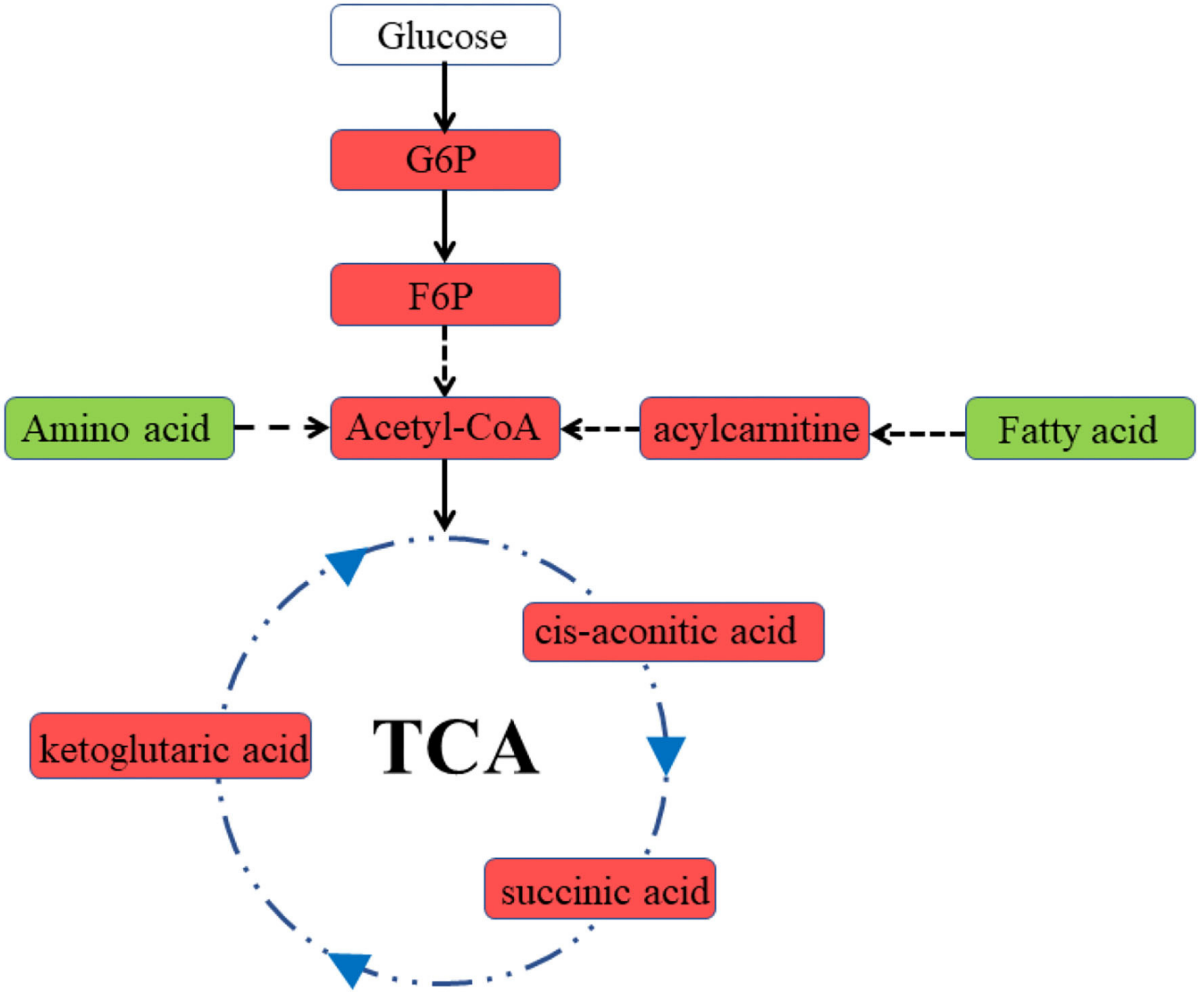
Diagram of cardiac metabolism in the bypass group. Red represents an increase in levels, green represents a decrease in levels. G6P, glucose 6-phosphate; F6P, fructose 6-phosphate.

Previous studies have demonstrated that fetal CPB may lead to cardiac insufficiency, which could persist for several hours after bypass (6). In the present study, we used the Tei index to assess cardiac function. The Tei index is not affected by ventricular geometry, heart rate, or gestational age and has good reliability and reproducibility for detecting fetal cardiac function (15). It is noteworthy that Tei can be greatly affected by altered loading conditions, so any measurement of Tei during the bypass process should be unreliable. The increase in the Tei indices suggested cardiac insufficiency in the bypass group. In addition, the heart rate of lambs receiving fetal CPB was significantly decreased, which may be associated with myocardial dysfunction. Consistent with the deterioration in cardiac function, significant metabolic changes occurred in the bypassed hearts. The heart is omnivorous. Immature hearts usually consume lactic acid and glucose, while mature ones prefer fatty acids (16). Unlike the general immature heart, increased use of multiple energy substrates, particularly fatty acids, was observed in the bypassed heart (Figure 6). Both lipid metabolism and mitochondrial metabolism were enhanced, indicating increased energy demand in the bypass group. Lipid metabolism displayed a higher yield of energy production but required more oxygen per ATP produced than glycolytic metabolism (17), which may aggravate myocardial hypoxia. Besides, increased lipid metabolism may lead to increased production of ROS and aggravate mitochondrial damage.

### Placental metabolism and placental function

Placental dysfunction is a major stumbling block in the development of fetal CPB (18). However, we did not observe typical placental dysfunction in the present study. There were no significant changes in umbilical artery PI and RI. The level of PCO_2_ in the bypass group was also not increased. The absence of placental dysfunction may be attributed to the short observation period and the use of membrane oxygenator. Notably, lactate levels in the bypass group elevated during bypass, indicating that the internal environment of these lambs was indeed disturbed. Correspondingly, there was little change in placental metabolomics. Nevertheless, some metabolic changes in the bypass placenta may indicate impending placental injury. Increased energy metabolism would improve oxygen consumption and exacerbate the workload of the placenta in gas exchange. Elevated levels of antioxidants (ascorbate palmitate, betaine) may be an adaptive compensatory response of the placenta to oxidative stress (19).

### Alterations in lipid metabolism

Changes in lipid metabolism occurred in many tissues of fetal sheep after bypass. In the heart, liver, kidney, and placenta, the metabolism of unsaturated fatty acids is altered; in the brain and placenta, the metabolism of phospholipids is altered. *In vivo*, Extracorporeal membrane oxygenation (ECMO) has been found to promote the oxidation of long-chain fatty acids in immature porcine hearts (20). In this study, we noted that CPB also promoted the oxidation of LCPUFAs, including arachidonic acid (ARA), DHA, and DPA, in fetal sheep. PUFAs are key components of cell membranes and precursors of some metabolites important for inflammation and oxidative stress (21). The deficiency of PUFAs can induce delayed neuronal cell migration and increase the risk of neurodevelopmental disorders (22). The deficiency of PUFAs may also contribute to neurodevelopmental disability in fetuses receiving cardiac surgery and warrants further study. Glycerophospholipids are an important component of neural membranes. They are involved in apoptosis, modulation of transporter activities, and the role of membrane enzymes (23). Significant alterations in the glycerophospholipid composition of neural membranes have been reported in neurological disorders (24). Alterations in multiple glycerophospholipids were observed in the bypassed brain, which may be associated with cerebral ischemia. In addition, lysophosphatidylcholine (LPC) has been reported to deliver LCPUFAs to the fetal circulation (25). A decrease in LPC in the placenta may aggravate the lack of LCPUFAs in the bypass group.

### Changes in amino acid metabolism

The main function of amino acids in the fetus is to provide raw materials for fetal growth and participate in oxidative reactions. Amino acid metabolism is accelerated when nutritional deficits are present (26). Significant changes in amino acid metabolism were observed in almost all tissues of the bypass group, mainly in decreased levels of glucose-producing amino acids. These findings are consistent with a previous clinical study in which various glycogenic amino acids were decreased in patients who underwent valve replacement under CPB (27). Davidson and colleagues also demonstrated a broad amino acid deficiency in neonates undergoing CPB by analyzing plasma metabolomics (35). The increased energy requirements of the fetal sheep in the bypass group led to accelerated amino acid decomposition, while excessive amino acid consumption may eventually lead to negative nitrogen balance and adverse outcomes. These findings suggest the possible need for early amino acid supplementation after fetal CPB. In addition, a decrease in homoarginine was found in all five tissues. Decreased homoarginine levels have been associated with endothelial dysfunction, cardiac insufficiency, ischemic encephalopathy, and intrauterine growth restriction (28). Previous studies have demonstrated arginine depletion after CPB (29), but this is the first time that abnormalities in homoarginine metabolism are observed, which may become a new marker of impaired organ function after CPB.

### Plasticizer metabolism

Di-(2-ethylhexyl) phthalate (DEHP) is a phthalate ester plasticizer commonly used in CPB tubing. DEHP has been reported to increase significantly in neonates receiving cardiac surgery (36). MEHP, a common metabolite of DEHP *in vivo*, was also found to accumulate in the blood and urine after CPB (30). In the present study, we observed increased levels of MEHP in the bypass group in the liver (136.7-fold of the control group, *P* = 0.00003) and brain (13.3-fold of the control group, *P* = 0.0007). DEHP exerts hepatotoxicity by disrupting redox homeostasis and inducing oxidative stress (31). DEHP is also harmful to the nervous system. DEHP exposure during pregnancy can alter the lipid metabolome in the fetal brain, leading to aberrant neurodevelopment (32). Since fetuses are less capable of metabolizing DEHP than infants, special tubing for fetal CPB are required to avoid plasticizer-related injuries. The DEHP leaching rate should be strictly limited, and plasticizers with lower toxicity and leaching rates should be developed.

### Limitations and future directions

There are also several limitations to the present study. First, cerebral function was not monitored, and blood biochemical indices don't appear to be appropriate for early assessment of liver or kidney function. Histology data and new biomarkers (like NGAL) may be more useful in assessing the function of these organs. Second, we could not make sure whether these functional and metabolic changes would persist after bypass. Further studies should be conducted over a more extended period in a larger cohort. In addition, each organ has various cell types and functional areas. Regional specificity for a particular pathway may exist in individual organs. In our analysis, we used only a small fraction of the entire organ sample. The application of spatial metabolomics may help reveal the complete metabolic landscape of the organs of fetal sheep receiving fetal CPB.

## Conclusion

This study performed a non-targeted metabolomic analysis of the fetal heart, liver, kidney, brain, and placenta in a fetal sheep CPB model. All tissues except the placenta displayed significant metabolic changes, and the heart displayed obvious function changes. Fetal CPB has common metabolic signatures in various tissues, including dysregulated lipid metabolism, altered amino acid metabolism, and accumulated plasticizer metabolites. These findings may strengthen the understanding of fetal bypass physiology and lay the foundation for specific organ protection in fetal cardiac surgery. Some of the results should also be applicable to postnatal cardiac surgery. Studies on substrate metabolism of immature myocardium may provide new ideas for neonatal cardiac protection. Obvious metabolic disorder in brain demonstrates the importance of brain function monitoring and cerebral protection. Dysregulated lipid metabolism and amino acid metabolism suggest possible need for early amino acid and PUFAs supplementation after bypass. Besides, Toxic plasticizers should be avoided in neonatal heart surgery. In conclusion, studying fetal metabolism, especially cardiac metabolism, is of great significance for the study of fetal CPB.

## Data availability statement

The raw data supporting the conclusions of this article will be made available by the authors, without undue reservation.

## Ethics statement

The animal study was reviewed and approved by Ethics Review Committee for Animal Experimentation of Guangdong Provincial People's Hospital.

## Author contributions

WW, XL, and CZ: concept and design. WW, YT, MT, HY, and JC: data analysis and interpretation. WW: drafting article. XL and CZ: critical revision of article. CZ: funding. BH and YD: data collection. HL: statistics. All authors contributed to the article and approved the submitted version.
